# Emotion Separation Is Completed Early and It Depends on Visual Field Presentation

**DOI:** 10.1371/journal.pone.0009790

**Published:** 2010-03-22

**Authors:** Lichan Liu, Andreas A. Ioannides

**Affiliations:** 1 Lab for Human Brain Dynamics, RIKEN Brain Science Institute, Wakoshi, Saitama, Japan; 2 Lab for Human Brain Dynamics, AAI Scientific Cultural Services Ltd., Nicosia, Cyprus; Macquarie University, Australia

## Abstract

It is now apparent that the visual system reacts to stimuli very fast, with many brain areas activated within 100 ms. It is, however, unclear how much detail is extracted about stimulus properties in the early stages of visual processing. Here, using magnetoencephalography we show that the visual system separates different facial expressions of emotion well within 100 ms after image onset, and that this separation is processed differently depending on where in the visual field the stimulus is presented. Seven right-handed males participated in a face affect recognition experiment in which they viewed happy, fearful and neutral faces. Blocks of images were shown either at the center or in one of the four quadrants of the visual field. For centrally presented faces, the emotions were separated fast, first in the right superior temporal sulcus (STS; 35–48 ms), followed by the right amygdala (57–64 ms) and medial pre-frontal cortex (83–96 ms). For faces presented in the periphery, the emotions were separated first in the ipsilateral amygdala and contralateral STS. We conclude that amygdala and STS likely play a different role in early visual processing, recruiting distinct neural networks for action: the amygdala alerts sub-cortical centers for appropriate autonomic system response for fight or flight decisions, while the STS facilitates more cognitive appraisal of situations and links appropriate cortical sites together. It is then likely that different problems may arise when either network fails to initiate or function properly.

## Introduction

Human social communication demands accurate judgment of others' disposition and intentions. Facial expressions are uniquely effective amongst the many clues that social encounters provide. In a typical social encounter, one must respond fast and accurately to subtle changes in the facial expressions of people around one. The need for accurate face affect recognition favoured the evolution of complex visual strategies recruiting a network of brain areas [1]–[3]. An often overlooked aspect of face affect recognition is its likely dependence on where the stimulus appears in the visual field. The fast decoding of emotional face expressions is of particular importance not only at the gaze center where attention is focused, but also in the upper visual field where the eyes of other faces within the immediate social group are likely to be located. The eyes of people close to us usually appear in the upper part of the visual field, and they carry strong clues about negative emotions (fear and anger) and surprise [4] – all emotions likely to require fast reaction. Moreover, as we covertly perceive people in our not so immediate environment, but still within a social gathering, faces are likely to appear in the upper part of the visual field. Here too we must respond to emotional expressions and gazes directed towards us, especially when they are negative, deciding in an instant whether to act, ignore or simply turn our gaze on them for a closer look. The lower visual field appears to have better spatial resolution than the upper [5], and is therefore more appropriate for the accurate response to positive emotion (happiness) [4], [6]. Happiness is more accurately expressed by the mouth, and it also requires reciprocation in social interactions, immediate for a person directly in our gaze (likely to be close to the centre of the visual field), but not necessarily with the utmost speed for a person a distance away (likely to be in the periphery of the visual field). It is thus evident that social demands may have influenced processing of facial expressions of emotion differently at the centre, upper and lower visual fields. The dependence of facial expression processing on the visual field is therefore plausible, but how might its anatomical and physiological support be implemented? The more frequent appearance of the faces of carers during infancy in the centre and upper visual fields may provide the driving force. The evolutionary pressure would have favoured selective pruning of connections supporting the analysis of the more frequently occurring encounters with the salient stimuli of the carer's face: the young infant must recognize the identity of the carer and establish with him/her an effective communication of affect very early, well before language develops. It is therefore plausible that early cortical plasticity would have differentiated anatomical connections in different parts of the visual field related to emotion recognition. These in turn would lead to differences in latency of activations or even the recruitment of different brain areas depending on where stimuli with facial expressions of emotion appear in the visual field.

The study of brain activity associated with the processing of facial expressions of emotion requires mapping activity across widely separated brain areas and accessing changes in the activity of these areas on a millisecond time scale. The required temporal resolution in human studies is only available using EEG and MEG. Both techniques provided evidence that face recognition is fast, beginning around or even before 100 ms post-stimulus [7]–[11]. Within 100 ms early category specific cortical activity was also identified [12]. As for the speed of face affect recognition, EEG evoked responses discriminated emotional from neutral faces around 120 ms [13]–[16], while event-related magnetic fields showed selectivity for emotion processing early, between 120 and 170 ms [17].

In the past, brain areas involved in recognition of facial expressions were localized with methods relying on slow hemodynamic responses at the expense of time resolution. EEG and MEG were considered poor for localization in general and specifically for deep structures such as the amygdale. Yet for some time now amygdala activations have been reconstructed from whole-head MEG data using distributed source analysis [18]–[20] or spatial filtering method [21]. In our earlier study of emotion processing we compared responses from normal and schizophrenic subjects. In normal subjects, but not in patients, we identified an early amygdala activity (30–40 ms) linked to the first strong peak in the primary visual cortex (70 ms) [22].

Most earlier studies on face affect recognition, including our own [20], [22], [23], used stimuli presented at the center of the visual field. These studies may therefore miss any dependence on visual field location of the underlying mechanisms of processing facial expressions of emotion. Recent studies have provided some support for the developmental and evolutionary arguments described above for a visual field dependence of recognition of facial expression of emotion [24]. A recent behavioral study has demonstrated that the same object (faces, mammals, body parts, objects, tools, vehicles) presented in different positions might evoke only partially overlapping or even completely distinct representations [25]. Using the same objects as in the behavioral study displayed at each of the four quadrants in an event-related fMRI study, the authors further showed the presence of position-dependent object representations in anterior regions of the ventral stream (lateral occipital complex and posterior fusiform sulcus) [26]. To date, only a limited number of neuroimaging studies have been systematically conducted using peripheral presentations to study the laterality effect on the processing of facial emotions. Even in these few studies, only left- and right-hemifield presentations were used [15], [27]–[29].

The main hypothesis tested in the present study is that brain activations elicited by faces with emotional expressions will depend on where stimuli are presented in the visual field. At the most general level we would then expect that the brain responses to different emotions will differentiate either in different areas and/or at different latencies, for stimulation in different parts of the visual field. Based on the earlier discussion of ecological and developmental factors we also hypothesize that stimuli in the upper visual fields would recruit the parts of the network that deliver fast autonomic response but do not necessarily reach consciousness early, while emotional faces presented in the lower visual field would recruit areas that are related more to accurate rather than fast evaluation. Emotional faces presented in the center of the visual field, where our immediate target of social interaction is likely to be, would recruit both sets of areas, i.e. areas like the amygdale that can lead to immediate autonomic response and other cortical areas capable of further cognitive elaboration of the input. Furthermore we hypothesized that control areas that are responsible for inhibiting areas with ultra fast responses would be preferentially active when fast and accurate responses are needed, specifically for faces presented at the center and upper visual field. In this work, we presented stimuli with facial expressions to one of the quadrants as well as the center to study laterality effects systematically. We recorded noninvasively from human brains millisecond-by-millisecond using a whole-head MEG system. This enabled us to capture the dynamic nature of the brain systems underlying the different facial expressions. Finally statistical parametric mapping of single timeslice, single trial tomographic estimates of activity identified significant changes in activity throughout the brain. This provided us with a model-independent way to examine the spatiotemporal dynamics differences between centrally and peripherally presented stimuli. We found fast emotion separation within 100 ms post-stimulus. Emotion separation emerged first at either the amygdala or STS, and the specific pattern and timing of this separation depended on where the faces appeared in the visual field.

## Materials and Methods

### Subjects

Seven healthy right-handed males (mean age 35, range 27–50) with normal or corrected-to-normal vision volunteered after giving their consent to take part in the study. The study was performed in accordance with the ethical standards laid down in the 1964 Declaration of Helsinki (The Code of Ethics of the World Medical Association). The RIKEN Research Ethical Committee approved the study.

### Stimuli

A face affect recognition task was used for the experiment. Stimuli were chosen from Ekman and Friesen's Pictures of Facial Affect [30]. Five actors (two male) whose expressions were best recognized in posing two facial emotional expressions (happy and fearful) and a neutral face were selected. In each recording run, 15 images (five actors × three emotions) were repeated once and a total of 30 images were randomly presented to subjects. The general visual qualities of each image were digitally reworked to ensure uniformity: a luminance meter was used to adjust the images to natural daylight conditions in rooms (average luminance of 30–40 cd/m^2^). Then all the images were mounted into the center of a mid grey background to ensure uniform figure/ground contrast.

### Experimental Design

We used a block design for presenting the images in different parts of the visual field: the images appeared at one of the five positions (center or quadrants) on the screen, fixed for each run. Each run consisted of 30 images on a gray background and 15 sec of the same background with a fixation cross before and after the 30 images. Hereafter the image position is referred to as CM (center middle), UL (upper left), UR (upper right), LL (lower left) and LR (lower right). At CM, images subtended 4° and 6° of visual angle horizontally and vertically. In each quadrant (UL, UR, LL, LR), images were 6×9° with an eccentricity of 10°. Each image was shown for 500 ms and 1 sec later an option list of the emotions was shown for 3 sec. Subjects had to name the emotion verbally as soon as the list appeared. The inter-trial interval was randomized between 1.5 and 2.2 sec. Three runs for each of the five image positions were recorded. The total recorded runs were therefore 15 (5 positions×3). The run order was randomized and counter-balanced across subjects. Two subject baseline runs were also recorded, one recorded before and the other after the task runs. In these two control runs, subjects were in place with the same luminosity and fixated on a cross as in the task runs.

### Monitoring Eye Movements

During the whole recording run, subjects fixated the center of the images for central presentation or a fixation cross at the screen center for peripheral presentation. To achieve this, one day before the main experiment, we trained subjects specifically to fixate centrally and not to look at the images directly when they appeared in the quadrants using the same experimental design as in the main experiment but with a different image set (JAFFE database) [31]. To monitor subjects' eye movements, we placed one pair of EOG electrodes 1 cm above and below the left eye (vertical movement) and another pair 1 cm lateral to the left and right outer canthus of the eyes (horizontal movement). We recorded and calibrated the EOG signal during training. On the experiment day, all subjects could perform the task without difficulty while maintaining central fixation as confirmed by the EOG recording. The full details of eye movement calibration procedure can be found elsewhere [32].

### MEG Signal Recording

We recorded MEG signals using a whole head Omega 151-channel system (CTF Systems Inc., Vancouver, BC, Canada) with additional electrodes monitoring artifacts from the subject's eye movements and heart function. The MEG signal was recorded in an epoch mode as a 5-second segment beginning from 500 ms before to 4.5 sec after each image onset. The recording was made with a low-pass filtering at 200 Hz and sampling at 625 Hz.

### MEG Signal Processing

Off-line, environmental noise was first removed from the MEG signal using the CTF software. The data were then filtered in the 3–200 Hz band. Note our low pass-band filter was set to 200 Hz, not up to 50 Hz (rarely 100 Hz) as in earlier EEG and MEG studies [10], [33]–[35]. This allowed us to maintain the dynamics in the recorded signals and enabled us to capture the fast responses in the signal that could have been eliminated by a narrow band-pass filter.

We then extracted trials from each run, 500 ms before to 1 sec after image onset. Careful off-line inspection ensured that the extracted MEG signal was free of contamination from subject's mouth movement during speech. Trials with blinks and eye movements (as indicated by the calibrated EOG signals) around image onset (−200 to 500 ms) were rejected manually. On average about 1–2 trials were rejected in some of the 15 task runs for each subject. For the remaining extracted data, we further removed subject's artifacts such as heart function and eye blinks and movements (not around image onset) using independent component analysis [36].

### Tomographic Analysis

We used magnetic field tomography (MFT) to extract tomographic estimates of activity from single trial MEG data. MFT is a non-linear method for solving the biomagnetic inverse problem. It produces probabilistic estimates for the non-silent primary current density vector **J** across the entire brain at each timeslice of the MEG signal [37]. MFT was first developed 20 years ago and over these years the method has been extensively tested with computer generated, phantom and real data including an fMRI/MEG validation study [38]. In some of these studies, we also used other source reconstruction methods for comparison with MFT [32], [38]–[40]. The key concept of MFT, specifically the fundamental difference between MFT and other linear methods like minimum-norm and spatial filter methods, is the use of a non-linear algorithm that can identify activity in single trials [18], [41], [42]. The specific form of non-linearity at the heart of the MFT algorithm has optimal stability and sensitivity and it is thus appropriate for localizing both distributed and focal sources without any prior assumptions about their number and form [40]. Applying MFT independently to each timeslice of data for each single trial allows us to do the post-MFT statistics across single trial subsets of each run, for each subject separately, utilizing the variance of the single trial responses. The MFT ability of mining the variance in the single trials endows the method with much higher sensitivity often allowing fine differences to emerge that escaped other source reconstruction methods [32].

Specifically in the present study, for each subject MFT was applied to each trial from the 15 task and 2 control runs, from 200 ms before to 600 ms after image onset at a step of 1.6 ms (total of 500 timeslices). For each timeslice, MFT produced an independent tomographic map of brain activity in a 17
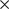
17
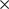
17 source space grid (grid-to-grid point separation was 8 mm) covering the whole brain.

### Post-MFT Statistical Parametric Mapping (SPM) Analysis

We applied our in-house SPM analysis to identify brain areas and latency periods when the activity was significantly different between the task and control runs [41]. For each grid point and at each time-slice, an unpaired t-test was used to test whether the two distributions were the same or not. We used the conservative Bonferroni adjustment to correct multiple grid-point comparisons. The statistical analysis makes no *a priori* assumptions about any regional activity or timing because it identifies loci of significant changes of activity in a model-independent manner: grid point-by-point statistical analysis throughout the entire brain was carried out for each timeslice. Specifically, the elements of the distribution were the smoothed values of the current density modulus within a moving window of 6.4 ms in a step of 3.2 ms. The resulting SPM maps for each contrast from each subject were then transformed to the Talairach space [43] and common changes in activity across subjects were identified.

### Regions of Interest and Activation Time Courses

Regions of interest (ROIs) were defined by location and direction. The location was based on consistent activations in the combined SPM maps across subjects. We identified ROIs in the early visual area (V1/V2), fusiform gyrus, amygdala, middle occipital gyrus (i.e. “occipital face area” (OFA)), STS, and medial prefrontal cortex (MPFC). The ROI locations were found to be very similar for central and quadrant presentations except for V1/V2 ROIs. For central presentations, four ROIs were defined for bilateral dorsal and ventral V1/V2 while for quadrant presentation, one ROI was defined for the activated part of the calcarine sulcus, e.g., for UL presentation, and only one V1/V2 ROI was defined for the right ventral area. Table 1 lists the coordinates of the ROIs and their corresponding brain structures.

**Table 1:** Talaraich (TAL) and Montreal Neurological Institute (MNI) coordinates in mm for the defined ROI centers averaged over the seven subjects for central presentation.

ROI	TAL	MNI	Brain structure	Other studies
**LV1/2D**	-10 -90 4	-6 -101 15	left calcarine gyrus 60% area 17 71% area 18	
**LV1/2V**	-9 -90 -10	-5 -99 -3	left calcarine gyrus 59% area 17 51% area 18	
**RV1/2D**	11 -88 7	13 -98 15	right calcarine gyrus 82% area 17 66% area 18	
**RV1/2V**	10 -88 -7	11 -94 -1	right calcarine gyrus 76% area 17 50% area 18	
**LFG**	-31 -56 -9	-33 -67 -8	left fusiform gyrus	TAL: -35 -63 -10 [49]
**RFG**	29 -56 -8	30 -67 -7	right fusiform gyrus	TAL: 40 -55 -10 [49]
**LAMY**	-21 -4 -24	-22 -9 -25	left amygdala 78% laterobasal complex	TAL: -20 -10 -28 [50]
**RAMY**	21 -2 -22	25 -5 -22	right amygdala 67% laterobasal complex	TAL: 20 -10 -30 [50]
**LOFA**	-32 -82 -1	-33 -95 6	left middle occipital gyrus	TAL: -30 -77 0 [47]
**ROFA**	32 -80 0	36 -94 4	right middle occipital gyrus	TAL: 31 -75 0 [47]
**LSTS**	-51 -56 14	-55 -65 22	left superior temporal sulcus	TAL: -55 -60 10 [52]
**RSTS**	50 -53 17	54 -65 22	right superior temporal sulcus	TAL: 52 -48 8 [52]
**MPFC**	-1 19 -13	-1 17 -14	medial prefrontal cortex	MNI: 0 15 -14 [51]

For quadrant presentations, only the definition for the V1/V2 ROI differed from that for central presentation, as listed in brackets. The corresponding brain structures for the ROIs are also listed in the rightmost column with the probability in different sections of early visual and amygdala areas, as obtained from the “SPM anatomy toolbox” [53].

The direction of ROI was defined using circular statistics: MFT produces probabilistic estimates for the non-silent primary current density vector 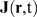 so the direction of 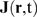 is essentially confined to two dimensions and its variation can be conveniently quantified and displayed using circular statistics [44], [45]. For each ROI at each presentation position, we applied the statistics to all the trials (e.g. 90 trials for central presentation) and obtained main directions for three time ranges (50–100 ms, 100–150 ms and 150–200 ms). If there was more than one main direction, then we chose the one that yielded the most consistent and strongest response across the trials as the main direction for that ROI. Thus for central and quadrant presentations, the location of ROI may be the same but the direction of the ROI was optimally defined for each image presentation position. Each ROI was defined as a sphere with a radius of 0.9 cm for V1/V2 or 1.0 cm for the rest of ROIs.

For each single trial, we calculated an ROI activation time course (ACV) 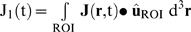 with 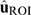 defined as the main direction of the ROI. We further applied analysis of variance (ANOVA, SPSS Inc., Chicago, Illinois, USA) to the ACVs to examine whether ROI activation patterns were significantly different between pairs of conditions. For example, for examining how the ROI activation was influenced by emotion, we applied ANOVA to ACVs using emotion (fearful, happy and neutral) as a fixed factor and subject as a random factor. At latencies when emotion became a significant factor, we further applied post-hoc two-tailed paired-samples t-test to examine which pairs of emotion were significantly different.

### Two Types of post-MFT Statistical Analysis and their Objectives

In summary, we used two types of post-MFT statistical analysis, each relying on a different property of **J**. First, the SPM analysis uses the modulus of **J**. By nature it is sensitive to robust changes in the energy content of the regional activation that are sufficiently high to stand out from the background level of activity. We used the SPM analysis to identify the loci of significant changes of activity. Second, the ROI time courses and the follow-up ANOVA rely on the current density vector of the regional activations. The dependence on the direction makes this second analysis more sensitive and hence capable of detecting changes in the organization of activity within an ROI, even when the overall change on the modulus (energy) is small. It is this second analysis that allowed us to identify the early regional activations and to determine their sensitivity to different facial expressions of emotion.

## Results

### Behavioral Results

**Figure 1:**
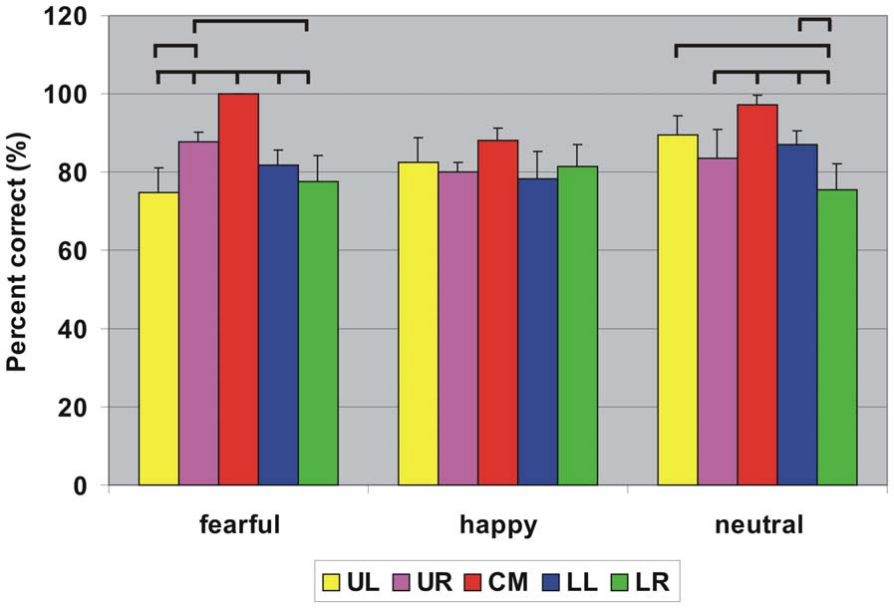
Subject's behavioral performance. Averaged percentage of correct trials over the seven subjects when the three facial expressions (fearful, happy and neutral) were presented at one of the five positions (CM, UL, UR, LL, LR). Error bar denotes 1 standard error. Horizontal bars indicate significant differences for comparisons between pairs of image position (p<0.05).

All seven subjects performed the task well above the chance level (33%). Performance was evaluated by the percentage of correct trials (%correct). Figure 1 compares the averaged-across-subjects performance at the five image presentation positions for each image type. Fearful faces were recognized best when presented at the center (p<0.002), at UR better than at UL (p<0.003), and at UR better than at LR (p<0.05). Likewise, neutral faces were significantly better recognized at the center than at other quadrants (p<0.04) except comparable with at UL, at UL better than at LR (p<0.0003), and at LL better than at LR (p<0.004). In contrast, there was no significant difference for happy faces placed at different positions. Our results are consistent with studies examining visual scanning patterns in healthy adults; these studies have indicated a left visual field bias and found that negative facial expressions elicit increased visual scanning in the right visual field [46].

To avoid confound effects caused by wrong judgment on the facial expressions, hereafter we will report results from correct trials only.

### MEG Signals

**Figure 2:**
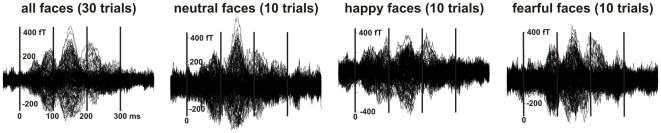
Typical signal waveform. Averaged MEG signals from one recording run when images were centrally presented in subject 1. The signal was averaged on image onset over 30 trials (all faces) or 10 trials (neutral, happy, fearful) faces in the run. All panels are shown in the same vertical scale.

Figure 2 shows typical MEG signal waveforms from one recording run when images were centrally presented in subject 1. This subject did not make any error in judging the facial expressions in this recording run, so the waveforms in Figure 2 were obtained from averaging over all faces (30 trials) or each facial expression (10 trials each). Although few trials were used in the averaging, the waveforms show in each case distinct peaks at about 40 ms, 80 ms, 140 ms and 210 ms.

### Regions of Interest

**Figure 3:**
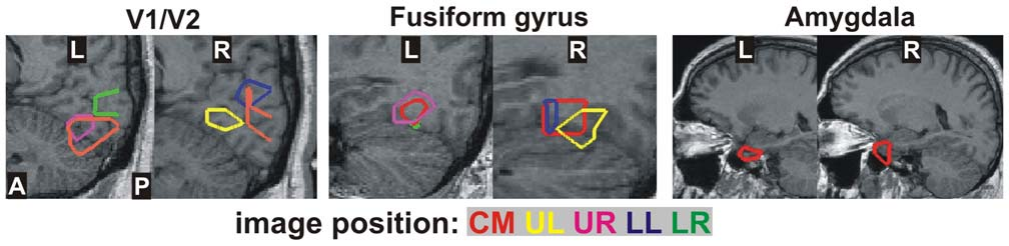
Common significant change of activity across subjects. The combined SPM maps are shown around bilateral V1/V2, fusiform and amygdala areas. The colors of the outlines represent the five image presentation positions.

To constrain the search for the effect of facial expression on brain activation, we first identified regions showing a visually evoked response to faces by comparing responses to faces against control stimuli (e.g. a fixation on a blank screen). After transformation of individual SPM maps into a Talairach space, we obtained common activated areas using a search radius of 1.0 cm (grid-by-grid point across the whole brain) and a search window of 19.2 ms (from 100 ms before to 500 ms after image onset with a step of 3.2 ms). Figure 3 shows some of the most consistently and significantly (p<0.05) activated areas in the combined SPM maps across subjects: bilateral V1/V2 (60–90 ms), fusiform gyrus (120–150 ms) and amygdala (130–140 ms; only seen for central presentation). These common activations were used to define the approximate areas for the ROIs in a model-independent way, i.e. data-driven and no *a priori* assumptions about the ROI locations. The ROI definition for each subject was then performed around the common areas by transforming to the individual subject MRI coordinates and using their own MFT solutions and post-MFT SPM results. The coordinates of the ROI centers averaged over subjects were listed in Table 1. The Talairach or MNI coordinates of our ROIs were similar to the coordinates reported in earlier fMRI studies using faces as stimuli, such as OFA [47], [48], “fusiform face area” [49], amygdala [50] and MPFC [51]. The STS identified in earlier studies covered a wide area; the variation may be due to different tasks and stimuli used in these studies. Using stimuli from the Pictures of Facial Affect, we identified similar activated STS area as in a fMRI study that faces displaying an emotional expression were compared with those displaying a neutral expression [52]. Furthermore, using “SPM anatomy toolbox” [53], we also obtained the probability in different sections of early visual and amygdala areas (Table 1). The V1/V2 ROIs mostly covered area 17 (V1) but also overlaped with area 18 (V2) [54]. The center of the amygdala ROIs was in the basolateral complex of the amygdala [55]. This section of the amygdala showed more sensitivity to facial expressions than gaze/head orientation, e.g. greater activation to threatening than appeasing facial expressions in awake macaques [56].

### Asymmetries in Visual Processing

**Figure 4:**
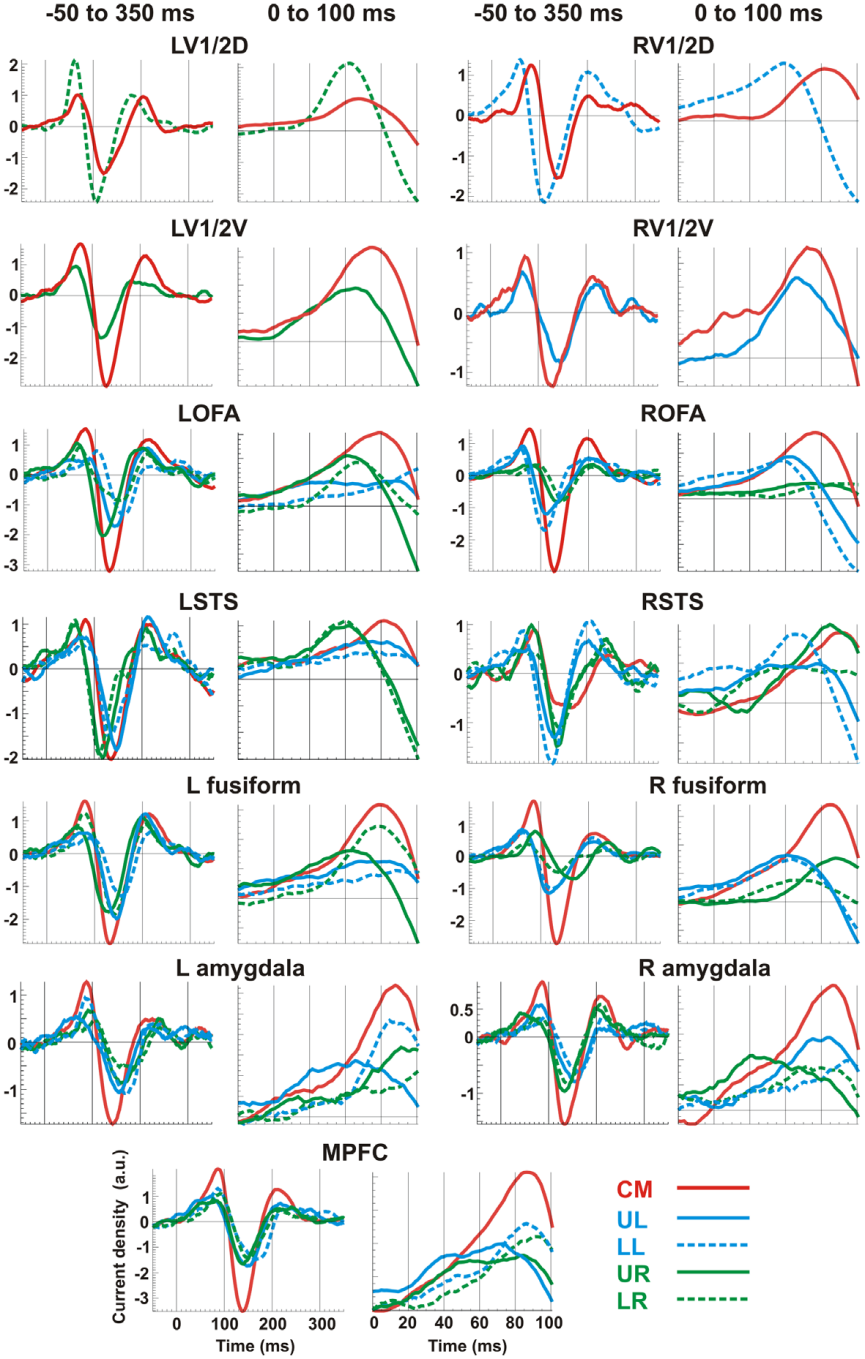
Regional activation time courses (ACV) for images presented at the five positions. Curves averaged on image onset across all seven subjects with a smoothing window of 20 ms. Odd columns are ACV plots for 50 ms before to 350 ms after image onset while even columns are the magnified view of odd columns (0 to 100 ms).

We constructed ROI activation time courses for each single trial from each task run in each subject. Figure 4 shows these time courses averaged across all trials and subjects for each image position with a smoothing window of 20 ms. Within the first 100 ms after image onset, by comparing the first peak of ROI activation (even columns in Figure 4), we observed that relative to the visual field where the image was presented, all ROIs except amygdala and MPFC activated earlier in the contralateral hemisphere. For example, the first peaks in left OFA for images presented in the right visual field (UR and LR, green lines) were earlier than those for images shown at the center (CM, red lines) and in the left visual field (UL and LL, blue lines). For amygdala, however, the ipsilateral hemisphere activated earlier than the contralateral hemisphere: The first peak in the left amygdala was at 76 ms and 96 ms respectively for images presented in the left and right visual fields, while in the right amygdala it was 90 ms and 66 ms respectively. For centrally presented images, the left and right amygdale first peaked at 88 and 86 ms respectively. To quantify the earlier ipsilateral hemisphere activation in amygdala, we gathered the data from each emotion and subject at each image position (i.e. 21 time courses for each of the five positions and 42 time courses for left and right visual fields) and applied ANOVA to the first peak latency using image position as a fixed factor and subject as a random factor, and found that amygdala activated significantly earlier in the ipsilateral hemisphere: p<0.021 for left amygdala and p<0.016 for right amygdala. An additional multiple comparison test (Scheffe post hoc test) showed that the first peak was significantly earlier for images presented in the ipsilateral visual field than at the center (p<0.0063 for left amygdala and p<0.0056 for right amygdala). For the MPFC, the first peak latency was more influenced by whether the images were presented in the upper or lower visual field (p<0.002). The latency was significantly shorter for upper visual field presentation than for central presentation (p<0.00001) and lower visual field presentation (p<0.003). Interestingly, upper-lower visual field presentation also influenced the first peak latency in fusiform, amygdala and OFA. The significance was p<0.003 for the left and p<0.002 for the right fusiform. In left fusiform, the first peak latency was significantly earlier for upper visual field presentation than for central (p<0.0003) and lower (p<0.00001) visual field presentation. In right fusiform, the first peak was significantly earlier for lower than for central (p<0.00001) and upper (p<0.0003) visual field presentation. Similarly for OFA, the left OFA activated earlier for upper than for lower field presentation (p<0.007) while the right OFA was earlier for lower than for upper field presentation (p<0.6, not significant). Both left and right amygdale activated earlier for upper than for central (p<0.02) and lower (p<0.4, not significant) field presentation.

### Emotion Separation

**Figure 5:**
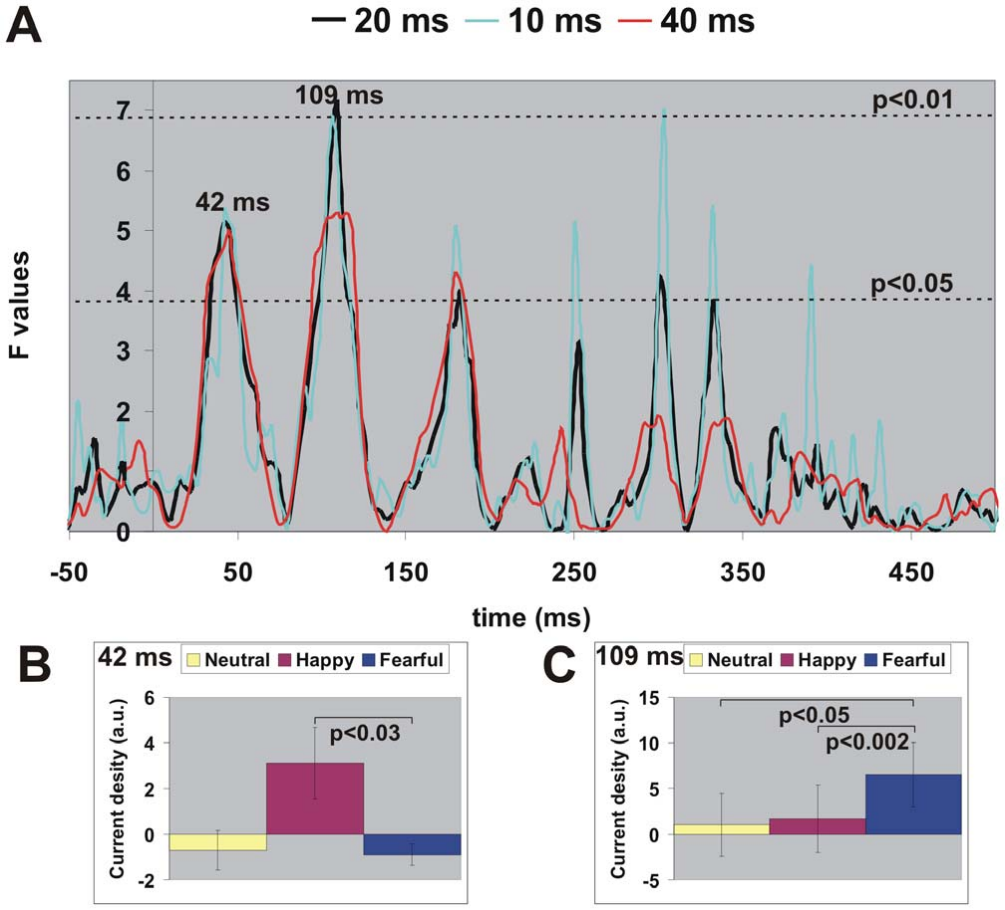
The calculation of latencies for emotion separation. (A) Effect of window length (10, 20 and 40 ms) on statistical significance (F values) from AVONA applied to right STS ROI time courses from centrally presented images. Dotted lines denote significance levels of p<0.05 and p<0.01. (B–C) Post-hoc T-test shows how the emotions are separated at the peak latencies in (A): 42 ms and 109 ms for ROI curves smoothed with a 20-ms window. Error bar denotes ±1 standard error.

We then examined how the ROI activation was influenced by emotion by applying ANOVA to ROI time courses using emotion (fearful, happy and neutral) as a fixed factor and subject as a random factor. At each image position (i.e. 21 time courses for each of the five positions and 42 time courses for left/right, and upper/lower-visual fields), we applied ANOVA to ROI time courses smoothed with sliding windows of 10, 20 and 40 ms. The results were similar for the three windows with the middle (20 ms) window yielding the best result. As an example using right STS activation curves from centrally presented images, the top panel of Figure 5 shows the effect of window length on statistical significance (F values, vertical axis): the F-curves peaked at similar latencies and as expected, the shorter the window (e.g. 10 ms), the more “spiky” (peaks) the F- curve, while the longer the window (e.g. 40 ms), the more smoothed the F-curve. The F-curves from the 20-ms window were most stable; this was also observed for all other ROI time courses. Thus hereafter we will report ANOVA results from ROI curves smoothed with the 20-ms window. For each ROI at each image position, we applied ANOVA from 200 ms before to 550 ms after image onset and will report latency ranges when emotion was a significant factor on the ROI curve, i.e., the F values were higher than the pre-stimulus period and passed the significant level of p<0.05 for at least two successive recording samples (6.4 ms), e.g. peak latencies around 42 ms and 109 ms in Figure 5A. Figure 5B–C show how the emotions were separated at these two latencies using post-hoc 2-tailed paired-samples t-test: at 42 ms, fearful faces were significantly different from happy faces (p<0.03) and again at 109 ms (p<0.002), when additional separation from neutral faces appeared (p<0.05).

**Figure 6:**
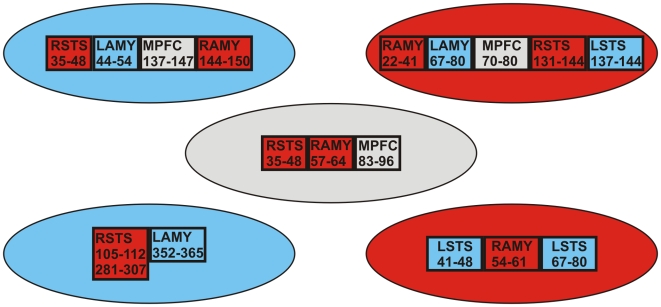
First time ranges for emotion separation in bilateral amygdala (AMY) and STS, and MPFC. The times are printed inside the boxes in millisecond. Images were presented at either the center or one of the quadrants. Gray, blue and red ovals represent fovea, left and right visual field presentation, respectively. In case of STS and AMY activations, boxes in the same color as the background oval denote ipsilateral activity while in different color denote contralateral activity.

For faces presented at the center or one of the quadrants, we identified all times in the range (0–550 ms) when ANOVA results showed emotion was a significant (p<0.05) factor on ROI activation time courses across seven subjects. In this paper we focused only on the first occurrences of emotion dependence. Figure 6 shows that within 100 ms after image onset, emotion separation was already established in STS, AMY and MPFC. For centrally presented faces, the emotions were separated first in the right STS (35–48 ms), followed by the right amygdala (57–64 ms) and MPFC (83–96 ms). After 100 ms, the separation was seen again in the right STS (99–115 ms), and extended to other areas such as left fusiform (102–108 ms; 140–153 ms), left V1/V2v (108–124 ms), right V1/V2v (147–157 ms) and right V1/V2d (166–182 ms). When images were presented in the periphery, emotion separation was seen first in the ipsilateral amygdala and contralateral STS. For instance, when images were shown in the upper-left visual field (Figure 6), the separation was first seen in right STS (35–48 ms) and then left amygdala (44–54 ms). Likewise, for images presented in the lower-right visual field (Figure 6), the separation showed in left STS (41–48 ms) and right amygdala (54–61 ms) with a re-activity in left STS seen later (67–80 ms). In the case of upper-right presentation (Figure 6), the separation appears in bilateral amygdala and STS: the responses are particularly fast in the amygdala (in right amygdala 22–42 ms and in left amygdala 67–80 ms), but rather late in STS (in left STS 137–144 ms and right STS 131–144 ms). The lower-left visual field showed emotion separation late: in right STS (105–112 ms; 281–307 ms) and in left amygdala (352–365 ms). Table 2 lists further in detail which pairs of expressions were separated in Figure 6. It is clear that within 100 ms post-stimulus emotions were separated for all except lower-left visual field presentations. Table 2 also shows that the happy expression appeared separating from the neutral and fearful expressions early.

**Table 2:** List of pairs of facial expressions showing early separations in the STS, amygdala and MPFC around 100 ms following images presented at the five visual field locations.

Visual field presentation	ROI	Separation of pairs of expressions	Time range (ms)
**CM**	RSTS	Happy-Fearful	35–48
	RAMY	Happy-Neutral	57–64
	MPFC	Happy-Fearful, Happy-Neutral	83–96
**UL**	RSTS	Happy-Neutral, Fearful-Neutral	35–48
	LAMY	Happy-Fearful, Happy-Neutral	44–54
**UR**	RAMY	Happy-Neutral, Fearful-Neutral	22–41
	LAMY	Happy-Neutral	67–80
	MPFC	Happy-Neutral	70–80
**LL**	RSTS	Happy-Neutral, Fearful-Neutral	105–112
**LR**	LSTS	Happy-Fearful	41–48
	LSTS	Fearful-Neutral	67–80
	RAMY	Happy-Neutral	54–61

## Discussion

In the present study we used MEG to map the spatiotemporal evolution of brain activity involved in a face affect recognition task, from superficial to deep areas, simultaneously at millisecond temporal resolution. This study is a continuation of our series of MEG studies on face processing using different types of visual objects including face stimuli and blurred images with normal [20], [23], [41], [57]–[59] and schizophrenic subjects [60], [61]. In one of these early studies we specifically examined emotion processing difference between normal and schizophrenic subjects, and we found that early amygdala activity (30–40 ms) was present but its linkage to the primary visual cortex (70 ms) was absent in patients [22]. In this 2002 study, we presented stimuli at central location only, under the implicit assumption that visual stimuli are processed largely in the same way wherever they are presented in the visual field. We examined the accuracy of this assumption in a series of MEG experiments by comparing brain responses to different types of stimuli presented at central and peripheral locations. The results using MFT analysis of average data showed that early V1 responses to small checkerboards [38] and full quadrant grating patterns [39] did not differ much for stimuli on the lower left and right quadrants, but both striate and extrastriate responses were faster for stimuli presented in the periphery as compared to the centre [58], [62], [63]. Laterality effects were further identified from the MFT analysis of responses elicited by faces [58], illusory figures [62], moving and stationary stimuli [64]. It thus became evident that to advance beyond our early studies on processing of facial expressions [22], [60], [61] we needed to analyze in detail the single-trial brain responses to stimuli with different facial expressions presented at different parts of the visual field. Given the limited time for an experiment (usually 4 hours before subjects become tired), it was impossible to include other control stimuli to identify face specific areas from separate runs or even to study the responses for all emotions in all quadrants. We therefore tested whether face specific areas could be identified in the same runs. This was indeed possible: using the same face stimuli as in the present study together with non-face stimuli (human hands and shoes), our recent study showed that the face-specific area, as defined by comparison between face and non-face stimuli, was also activated when faces were compared with baseline runs (simple fixation), or when faces at post-stimulus period was compared with pre-stimulus period [65]. The present study is therefore founded on these earlier results: we used a subset of the face stimuli from our earlier studies (present study: three categories only – neutral, happy and fearful faces; earlier studies [20], [23], [41], [57]–[59]: seven categories – neutral and emotional faces showing six basic emotions). The use of fewer categories and conditions allowed us to build a strong statistical basis and to focus our investigation on addressing the following questions: how early the amygdale and other areas are activated, how their activation depends on where the stimulus appears in the visual field, and whether emotions are separated in the same or different areas. We found that for centrally presented faces, the emotions were separated fast, within 100 ms post-stimulus, first in the right STS (35–48 ms), followed by the right amygdala (57–64 ms) and MPFC (83–96 ms). In comparison, when images were presented in one of the quadrants, emotion separation first appeared in the contralateral STS and ipsilateral amygdala.

The results of our analysis supported both the main hypothesis and the more detailed predictions. In summary we found clear evidence that brain activations elicited by facial expressions depend on where stimuli are presented in the visual field. This dependence produced a systematic differentiation of emotions that was identified first in the STS or amygdale. Our results also supported the specific hypotheses we made based on ecological and developmental arguments: the responses to stimuli presented in the upper visual field separated according to emotions early in the amygdala, a key part of the neural network for fast, probably unconscious, autonomic response. Stimuli in the lower visual fields differentiated emotions first in the contralateral STS, a brain region that was regarded having a primary role in perception of dynamic facial features such as expression, eye gaze and lip movement [1]. Again as predicted, stimuli presented at the centre of the visual field separated emotions fast in both the cognitive (STS) and autonomic (amygdale) brain areas. Finally emotion separation in the MPFC was identified soon after it appeared in the amygdala for stimuli presented at the centre and in the upper visual field, consistent with the role of this area as a monitoring area for the early amygdala activity, i.e. capable of exerting inhibitory influence if necessary on the amygdala output. Next we describe in turn the main findings for the activations in the key areas – amygdale, STS and MPFC.

### Amygdala

The amygdaloid complex is known for its role in the processing of emotion [66]. Here, the combined SPM results across the subjects showed that the amygdala activated robustly above baseline around 140 ms, but only for central presentation (Figure 3). This implies that amygdala activations were more robust and/or more time-locked to stimulus onset for stimuli presented centrally than peripherally (Figure 4). The second type of post-MFT statistical analysis identified fast amygdala activity within 100 ms, and showed that the amygdala activation patterns depended on where the stimuli were presented (Figure 4): when images were presented in the periphery, the amygdala on the ipsilateral side of the presentation activated significantly earlier than the one on the contralateral side. This ipsilateral response was also faster than that from central presentation. The earlier ipsilateral activation is unique for the amygdala among all the areas that we have studied in the present study.

The amygdala is established as an important area for emotional processing. However, it is still under debate whether amygdala activations are specific to any emotion or not. Many human neuroimaging studies have shown that amygdala involvement in processing of emotional stimuli that is more related to negative affect or withdrawal [67]. Some recent studies also suggest that the amygdala activations are nonspecific to any emotion, such as in a 4T fMRI study, Fitzgerald et al. [68] showed that the (left) amygdala was activated by each of the six facial expressions separately and its response was not selective for any particular emotion category. In the present study we found that the amygdala was one of the key areas (but not the only one) in emotion separation, especially in the early time interval (well within 100 ms for all except the lower-left presentation). For each peripheral presentation of faces, emotional expression was separated first in the ipsilateral amygdala (Figure 6). For central presentation, emotion separation was seen in the early latencies of 57–64 ms, in the right amygdala. This may be related to the larger volume of the right amygdala, particularly in right handed subjects [69].

### Superior Temporal Sulcus

Human neuroimaging studies have also implicated the STS in perception of dynamic facial features, such as expression [70]–[72] and direction of eye gaze [73]–[79]. In a recent fMRI study, Engell and Haxby [52] further compared the responses within the right STS and revealed that expression and averted-gaze activated distinct, though overlapping, regions of cortex. In the present study, we found the STS activation patterns were more affected by the left-right than the upper-lower visual field presentation (Figure 4): for quadrant presentations the contralateral STS activated earlier than the ipsilateral STS, while for central presentation, the left STS was earlier than the right STS (82 ms vs. 93 ms). Regarding emotion separation, the STS was involved fast: within 50 ms in the contralateral STS for UL and LR presentations and in right STS for central presentation, within 150 ms in the bilateral STS for UR, and within 120 ms in the contralateral STS for LL presentation.

### Medial Prefrontal Cortex

Animal studies have shown projections from the amygdala to the basoventral and mediodorsal prefrontal regions in rhesus monkeys [80], and anatomical connection between the basolateral amygdala and the MPFC in rats [81]. Additionally, electrolytic lesions of the ventral but not the dorsal MPFC interface with the extinction of Pavlovian conditioned freezing in the rats [82]. The effective amygdala connectivity was studied in a recent meta-analysis of human fMRI data using structural equation modeling constrained by known anatomical connectivity in the macaque [51]. One of the strongest bi-directional links identified was between the amygdala and MPFC (subgenual cingulated, BA25). MPFC is proposed to exert an inhibitory, top-down control of amygdala function [83], resulting in contextually appropriate emotional responses [84], [85]. A recent lesion study showed that emotion recognition was impaired following ventromedial, but not dorsal or lateral, prefrontal damage [86]. In the present study, we also observed the MPFC activations following emotional faces onset and found that their patterns were more influenced by upper-lower than left-right visual field presentation (Figure 4): the first peak latencies were significantly earlier for upper than lower/central visual field. MPFC was also seen in emotion separation, for upper and central visual field presentations only, and tended to be after the separation in the amygdala. Our results support the idea that MPFC receives input from the amygdala and then influences the emotional processing when fast responses are required, for example, when a threat appears in the upper visual field demanding immediate defensive action. We note that the MPFC activation identified in our study was earlier in time and more medial and posterior in location than two earlier MEG studies [21], [87]. In terms of location it corresponded best to the subgenual location of maximal connectivity with the amygdala identified in the recent human fMRI meta-analysis [51].

### Early Responses and Emotion Separation in Higher Order Brain Areas

There are two contrasting views that are often expressed with some conviction by their respective supporters. One view claims that there can't be early responses in “higher-order” areas such as the amygdale. If a response is demonstrated within 100 ms, then the claim softens to a statement that the corresponding neural activity is non-specific, i.e. it carries no information about face identity or emotion [88]. The opposing view suggests the existence of rapid pathways associated with “higher-order” processing of information conveyed by socially relevant stimuli. According to this view, another path is present – it can carry the signal to key brain areas bypassing V1, and also allows stimuli with social or survival information to be quickly recognized in extra-striate areas and especially the amygdale [89]–[91].

In the present study we made no *a priori* assumptions about which of the above two views might be right. We designed the experiment to test in a straightforward manner how activity spreads and how emotion may differ for stimuli presented at varied parts of the visual field. Our data-driven approach is an objective way to search which, if any, amongst many possibilities is supported by the data. We avoided masking because we did not want the effect of more than one stimulus onset and offset to be present. We did not use large images that would excite different parts of V1 that might interfere with each other. The results reported here show clear evidence for early activation in “higher-order” areas, i.e. the STS, amygdale and MPFC activated well within 100 ms post-stimulus (Figure 4). Our results thus support the view of separate and rapid pathways for visual processing. Further, the separation of emotions also occurred in these areas early, again well within 100 ms for all except the lower-left presentation. This suggests that the fast pathway leading to the amygdale and MPFC is capable of fair analysis of the stimulus, not merely the detection of its presence or absence.

### Happy Advantage

Our results show a trend of happy facial expression separating from neutral and fearful expressions early (Table 2). For example, within 100ms after the stimuli presented at the centre, the happy face was first separated from the fearful face in the right STS around 42 ms, followed by a separation of the happy face from the neutral face in the right amygdala around 60 ms, and then separations of happy-fearful and happy-neutral faces in the MPFC around 90 ms. This early separation of happy facial expression from other expressions is also observed for stimuli presented in other four quadrants (Table 2). It is tempting to link our results to “happy advantage” – faster recognition of happy facial expression compared to sad and disgusted facial expressions as demonstrated in a behavioral study [92]. This behavioral study further showed that the happy advantage was preserved when low-level physical differences between positive and negative facial expressions were controlled by using schematic faces, and the effect was not be attributed to a single feature in the happy faces (e.g. up-turned mouth line) [92]. Although our results cannot rule out that the early emotion separations are driven by the same low level properties as the ones governing early visual processing via V1, the early latency of the separation and the “happy advantage” point to an alternative direction. Likely the primitives that drive early emotion separation are more complex physical features of the stimulus, or a collection of them is more easily associated with how emotion is expressed in a face.

### Asymmetries in Visual Processing: their Significance and What might Drive them

Our results reveal clear asymmetries in visual processing: the earlier activation on the contralateral hemisphere for cortical activations in the occipital areas, including the STS, is well established. We further show two novel and potentially important asymmetries in visual processing: the earlier amygdale activation on the ipsilateral side for peripheral presentations, and the earlier MPFC activation for upper visual field presentation as compared to central and lower visual field presentations. These findings demonstrate that the almost automatic association of visual field presentation and contralateral hemisphere activation is only partially valid [93]: left-right visual field asymmetries should not be automatically interpreted in terms of hemispheric specialization alone [94].

Likewise, it is often claimed that the emotional expression of faces presented in the left visual field are recognized better because they are processed in the right hemisphere, but the evidence is rather mixed [95]. Our results show some biases exist, e.g. ipsilateral bias in amygdale, contralateral bias in occipital cortical areas, and upper-lower bias in MPFC. It is therefore likely that left-right visual field asymmetries identified in experiments sensitive to occipital-temporal cortical areas would appear differently in other experiments sensitive to frontal lobe activity, as if the visual world has been twisted by 90 degrees. This situation is reminiscent of Bryden and Underwood's comment [96] on Previc's account of upper versus lower visual field specialization: the upper and lower visual fields are strongly associated with far versus near vision, respectively, giving rise to clear ecological differences in the types of information that are typically encountered in the upper versus lower fields [97].

What might drive these asymmetries and how such high level primitives may have been selected? We have already commented in the Introduction that, during infancy, evolutionary pressure would have favored selective pruning of connections supporting the analysis of the more frequently occurring encounters with the salient stimuli of a carer's face. We could then attribute the effective separation of emotions when stimuli are presented in the upper right quadrant to the tendency of leftward bias when holding a newborn young infant. A leftward held infant will have the face of its mother in the upper right quadrant of the visual field (when the eyes are in the most comfortable gaze position) [98]. The tendency to hold infants on the left has been attributed to many factors including emotional communication between infant and mother [99]. A recent study [100] reported three results: (a) mothers displayed a significant leftward (71%) holding bias, (b) mothers with affective symptoms held their babies more on the right and more frequently in the vertical position, and (c) hemispheric specialization for perceiving visual emotions had no significant effect on the holding-side biases of new mothers. The above results fit our proposed framework: holding a baby on the left exposes the mother's face in the upper right visual field, the quadrant entrusted by evolution to be critical for emotion separation, likely through preserving ipsilateral connection to the right amygdale. A mother with affective symptoms is likely to have inappropriate facial expressions, and thus may adopt some evolutionary sensible strategy to avoid her face being exposed (e.g. vertical holding) or being seen in the upper right visual field of the infant. Further, the hemispheric specialization for perceiving visual emotions of the mother is not relevant because the process is optimized for the training of the infant's rather than the mother's neural machinery for emotion recognition.

On the basis of our results, we propose that the fast sub-cortical pathway to the amygdale may rely on complex primitives that separate different facial expressions precisely, because the original pruning was done during infancy according to the conjunction of circumstances (hunger, pain, comfort etc.) and the facial expressions of the mother or carer.

### Consistency of Early Emotion Discrimination in Brain Activations and Behavior

The early emotion-related brain responses (well within 100 ms after image onset) are likely related to unconscious processes and it is therefore risky to use them for extrapolating to behavior (at least 1.5 sec after image onset in the present study). It is nevertheless worth commenting on the consistency between the behavioral and brain imaging results, at least for the cases where emotion separation is extreme. For central presentation, good performance is expected and the presence of early emotion separation in the right STS is likely to be related to an early influence of conscious perception. The early separation of responses to different emotions in the right amygdala is likely to be related to early preparation for action, which nevertheless may be further controlled by the MPFC where emotions are separated some 30 ms after the right amygdala. For stimuli presented in the periphery, the fastest emotion separation is identified in the right amygdala for presentations in the upper right visual field. This is consistent with the best performance for recognition of fearful facial expressions in the same quadrant (Figure 1). Presentations in the lower left visual field are the only case in the periphery where no emotion separation is achieved in either amygdale or STS within 100 ms; this relatively slow development of emotion sensitivity is not associated with obvious significant deterioration of accuracy in our behavioral results (Figure 1). We emphasize that our present study was not designed to link brain activations with behavior directly, so the above rather speculative statements are simply flagged as possible targets for future studies to clarify.

The results of our tomographic analysis of brain activity are also consistent with earlier behavioral studies, providing what we believe are the first links between neuroscience and psychological explanations. We have already commented about the consistency of the specific emotion separations we have found and the “happy advantage” reported in the literature [92]. Extreme differences between upper right and lower left visual quadrants have been reported in the rather different context of visual search (visual search asymmetries in three-dimensional space) [101]. This study reported that performance in the lower left visual field was slower and least accurate. In contrast a reaction time advantage was found for upper versus lower and right versus left visual field.

### Impact of the Results on Theories of Face and Emotional Expression Processing

There is strong evidence that recognizing a face and its emotional expression are achieved by parallel processes that proceed fairly independently of each other [102]. Furthermore, emotional content can modulate early processing, possibly via separate cortical [19], [87] and sub-cortical pathways [21], [103], [104] with the emphasis shifting from specialized areas to a distributed processing [2], [105]. However, the independence of face recognition and facial expression recognition is not complete. Under special conditions an asymmetric interaction has been reported between different aspects of face perception: irrelevant variations in facial expression or facial speech do not influence reaction times in a face identity task, while irrelevant variations in facial identity do influence performance in a facial expression classification task [106]. A plausible synthesis of the evidence so far is that processing of identity and emotional expression proceed largely independently in the early stages of processing, with a fast network of sub-cortical pathways specialized for processing biologically salient features in general and facial emotional expression in particular. The influence of identity on facial expressions would occur later in time (usually after 100 ms) as compared to the early brain responses and emotion separations (within 100 ms) that we reported here, a postulate that can be easily tested in future experiments.

Earlier studies assumed a largely visual field independent network structure, and the few that employed techniques with fine temporal resolution have either used low pass data, e.g. below 20 Hz [19], [87] or long analysis windows [21]. In the present study we have exploited the high temporal resolution of MEG, maintained the dynamics in the recorded signals, and applied the source reconstruction directly to single trial data in each subject. Most importantly, our study examined separately the processing of facial emotional expression when stimuli were placed in each quadrant and the center of the visual field. The statistical analysis across trials and subjects demonstrated that separating the facial emotional expressions was completed extremely fast, within 100 ms post-stimulus. This separation was first achieved either in the contralateral STS or ipsilateral amygdala depending on where the stimulus was presented in the periphery, or nearly simultaneously in the right STS and right amygdala and a little later in the MPFC when stimuli were presented at the center of the visual field. Earlier reports on top-down facilitation of visual recognition are probably related to slower processes arising from conscious recognition [87]. The new results reported here reveal an earlier stage of processing that is fragmented according to its origin in different parts of the visual field. It is therefore to be expected that this early and fragmented stage of processing does not reach consciousness. We nevertheless anticipate that the integrity of these early processes plays an important role in our daily interactions, influencing our own subtle facial expressions and voice intonation and thus determining our social persona. It is also likely that failure in one or more of these pre-conscious fragments would lead to specific pathologies.
